# Cochlioquinones 1968–2024: Chemistry, Biosynthesis, and Biological Activities with Future Perspectives

**DOI:** 10.3390/jof11100712

**Published:** 2025-09-30

**Authors:** Huiqi Fang, Qi Li, Lin Chen, Gang Ding

**Affiliations:** 1 State Key Laboratory of Bioactive Substance and Function of Natural Medicines, Institute of Medicinal Plant Development, Chinese Academy of Medical Sciences and Peking Union Medical College, Beijing 100193, China; klxsxjsw@163.com (H.F.); liqicpu@163.com (Q.L.); 2 Henan Engineering Research Center of Chemistry and Biology of Medicinal Resources, Zhengzhou 450063, China; 3 Henan Comprehensive Utilization of Edible and Medicinal Plant Resources Engineering Technology Research Center, Zhengzhou 450063, China; 4 Zhengzhou Key Laboratory of Synthetic Biology of Natural Products, Huanghe Science and Technology College, Zhengzhou 450063, China; 5 Henan Joint International Research Laboratory of Drug Discovery of Small Molecules, Huanghe Science and Technology College, Zhengzhou 450063, China

**Keywords:** meroterpenoids, cochlioquinone, structures, NMR features, bioactivities, biosynthesis

## Abstract

Cochlioquinones are a member of meroterpenoids possessing a core 6/6/6/6 tetracyclic ring system, which originate from the polyketide-terpenoid hybrid biosynthesis. Up to date, there are eighty-one analogues with diverse post-modifications isolated from different fungi, most of which exhibit different biological activities, such as phytotoxic, antibacterial, cytotoxic, and immunosuppressive effects. Structurally, cochlioquinones can be mainly categorized into two classes: benzoquinone-type and phenol-type cochlioquinones, respectively. In this review, chemistry and biology of cochlioquinones including the structures, NMR and MS features, bioactivities, and biosynthesis from 1968 to 2024 are systematically summarized, which might provide insights into the exploration and utilization of this group of meroterpenoids in the agricultural or pharmaceutical industry.

## 1. Introduction

Meroterpenoids were first termed by Cornforth in 1968 to describe a class of secondary metabolites derived from hybrid terpenoid biosynthetic pathways [1]. These compounds are widely distributed in plants, animals, fungi, and bacteria, and exhibit broad and remarkable biological and pharmacological activities. Representative examples include chlorophyll, ubiquinone-10, vinblastine, and vitamin E, among others [2,3]. Moreover, the majority of meroterpenoids are of fungal origin, and some have been developed into clinical drugs or prodrugs, such as the immunosuppressant mycophenolic acid, the antimicrobial and anti-angiogenic agent fumagillin, and the acetylcholinesterase inhibitor terreulactone B [4].

Based on differences in their biosynthetic pathways, the structures of fungal meroterpenoids can be divided into two characteristic moieties: a terpenoid part (mostly originating from the mevalonic acid pathway) and a non-terpenoid part (derived from polyketide, amino acid, shikimate, or hybrid biosynthetic pathways) [5,6,7,8,9]. Among these, polyketide-terpenoids constitute the majority. Depending on the number of polyketide extension units in the biosynthetic pathway, polyketide-terpenoids can be classified into three categories: triketide-terpenoids, tetraketide-terpenoids, and other polyketide-terpenoids [10,11].

**Figure 1:**
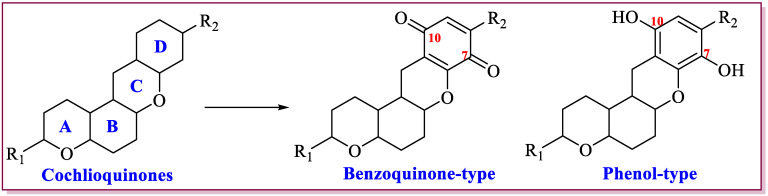
Classification of cochlioquinones.

Cochlioquinones are a member of meroterpenoids, which was first found from red clover pathogen causing leaf spot diseases. Recently, numerous studies confirmed that different plant pathogens and endophytic fungi could produce cochlioquinones [12,13,14,15,16,17,18,19,20,21,22,23,24,25,26,27,28,29,30,31,32,33,34,35,36,37,38,39,40,41,42,43,44,45,46,47,48]. This group of meroterpenoids are polyketide-terpenoid hybrid molecules, featuring a core 6/6/6/6 tetracyclic ring system consisting of a sesquiterpenoid part and a polyketide part. Different post-modifications including esterification, oxidation, hetero cyclization, and also rearrangement and spontaneous oxidation, which leads to formation of diverse cochlioquinone analogues [12,13,14,15,16,17,18,19,20,21,22,23,24,25,26,27,28,29,30,31,32,33,34,35,36,37,38,39,40,41,42,43,44,45,46,47,48]. Up to date, there are eighty-one analogues isolated from different fungi. According to structural features at the polyketide part, cochlioquinones can be mainly categorized into two classes: benzoquinone-type cochlioquinones (with carbonyl group at C-7 or C-10) and phenol-type cochlioquinones (with hydroxyl group at C-7 or C-10), respectively (Figure 1).

Functionally, cochlioquinones are recognized as fungal phytotoxins, with certain analogues demonstrating antibacterial, adjuvant, and pharmacological properties. It should be noted that cochlioquinones are regarded as phytotoxins (fungal metabolites toxic to plants) rather than mycotoxins (toxic to humans and animals), a distinction underscored by the current lack of toxicological data implicating them in food safety concerns. Beyond these roles, cochlioquinones exhibit a broad spectrum of bioactivities including phytotoxic, cytotoxic, and immunosuppressive effects, although their mechanisms of action and structure–activity relationships (SARs) remain incompletely elucidated. Isotopic tracer studies provided initial insights into their biosynthesis [49,50,51], and recent work by Ge’s group has further clarified the pathway using bioinformatic, chemical, and genetic approaches [29].

While several reviews have summarized the structures and bioactivities of meroterpenoids in general [1,4], a dedicated analysis focusing on the chemistry and biology of cochlioquinones has been lacking. This review systematically summarizes research on cochlioquinones from 1968 to 2024 (Table 1), aiming to facilitate future exploration and potential applications of these compounds in agricultural and pharmaceutical contexts.

## 2. Benzoquinone-Type Cochlioquinones

**Figure 2:**
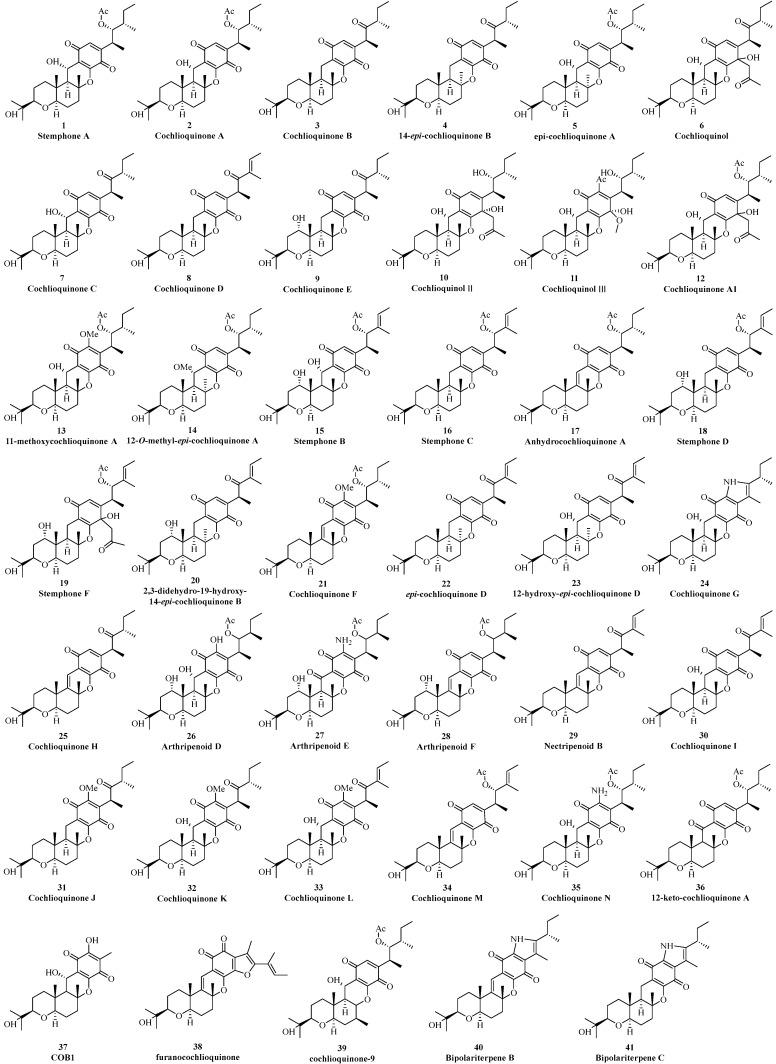
Benzoquinone-type cochlioquinones.

Thus far, forty-one benzoquinone-type cochlioquinones have been reported from different fungi (Figure 2). In 1968, Lawrence et al. isolated the first cochlioquinone stemphone (**1**) from the pathogenic fungus *Stemphylium sarcinaeforme* without stereochemistry determination, and its absolute configuration was later established by single crystal experiment [12]. Two yellow pigment-like substances cochlioquinone A (**2**) and cochlioquinone B (**3**) were isolated from the mycelia of the plant pathogenic fungus *Cochliobolus miyabeanus* in 1971, and their structures were determined systematically based NMR, X-ray single crystal diffraction experiments and biosynthetic analysis [13]. The known compounds stemphone (**1**) and cochlioquinone A (**2**) were later also isolated from the pathogenic fungus *Drechslera sacchari* [14]. 14-*epi*-cochlioquinone B (**4**) was purified from the fermentation broth of *Neobulgaria pura* in 1994, and its planar structure was same as that of cochlioquinone B (**3**) but with different orientation of 26-Me based on ROESY analysis [15]. A new cholesterol acyltransferase inhibitor, *epi*-cochlioquinone A (**5**), was separated from the soil fungus *Stachybotrys bisbyi* SANK 17777, and its ^1^H-NMR spectrum was near the same as that of cochlioquinone A (**2**) except the chemical shift value of 26-Me (*δ*_H_ 0.64) in a higher field than that of **2** (*δ*_H_ 1.02), indicating that it was the stereoisomer of cochlioquinone A (**5**) at C-14 and this postulation was confirmed through single crystal diffraction experiment [16]. A new phytotoxin, cochlioquinol (**6**), was isolated from the plant pathogenic fungus *Bipolaris cynodont* in 1996, and its structure was suggested to be the reduction product at C-7 through NMR spectral analysis, which was further supported by single crystal diffraction [17]. Based on the bioactivity-guided method of plant toxicity, a series of cochlioquinones including five new benzoquinone types, namely cochlioquinones C-E (**7**–**9**), cochlioquinol II (**10**), and cochlioquinol III (**11**) were separated from the pathogenic fungus *B. cynodontis cynA*. The relative configuration of these new compounds (**7**–**11**) was determined by NMR analysis, and their absolute configurations were suggested to be same as that of cochlioquinone A (**2**) considering the same biosynthetic pathway [18].

In 2003, a new anti-angiogenic agent, cochlioquinone A1 (**12**), was isolated from the pathogenic fungus *B. zeicola* [19]. Its NMR data closely resembled those of **2** except that the carbonyl group at C-7 was reduced to a hydroxyl group together with addition of an acetylmethyl group same as that found in cochlioquinol (**6**). 11-methoxycochlioquinone A (**13**) and 12-*O*-methyl-*epi*-cochlioquinone A (**14**) were purified from *B. zeicola* and *S. chartarum* through bioactivity-guided method [20]. These two new analogues (**13** and **14**) were similar to **2** and **5** by possessing an additional methoxy signal at C-11 and C-12, respectively, which were confirmed by NMR experiments [20]. Two new cochlioquinones stephones B and C (**15** and **16**) were isolated in 2005 by Omura’s group, and their structures possessed a double bond at C-2/C-3 according to NMR data analysis [21]. A new anti-cancer metabolite anhydrochlioquinone A (**17**) was found in *B. oryzae*, which might be originated from cochlioquinone A (**2**) by the loss of one molecule of H_2_O to form the double bond at C-12/C-13 [22].

In 2008, Tomoda et al. isolated two new cochlioquinones named stephones D (**18**) and F (**19**) from the fermentation broth of *Aspergillus* sp. FKI-2136 based on antagonistic experiment, and their structures were analyzed through two-dimensional NMR spectral analysis [23]. Wang’s group isolated 2,3-dihydro-19α-hydroxy-14-*epi* cochlioquinone B (**20**) from the endophytic fungus *Nigespora* sp. MA75 inhabiting the mangrove plant *Pongamia pinata* in 2012. The planar and relative configuration of **20** was mainly determined by NMR data analysis, but the absolute configuration at C-19 was not solved as Mosher’s reactions were not successful [24]. The known compound anhydrochlioquinone A (**17**) and its new analogue cochlioquinone F (**21**) was separated from the plant pathogenic fungus *B. luttrelli* [25]. Based on the same biosynthetic pathway, the configuration of **21** was suggested to be same as that of **17** [25].

In 2015, Hashimoto separated two new metabolites *epi*-cochlioquinone D (**22**) and 12-α-hydroxy-*epi*-cochlioquinone D (**23**) from the fungus *Helminthosporium velutinum* TS28 through anti-fungal *C. miyabeanus* growth screening experiments [26]. The relative configurations of these new compounds were analyzed by coupling constants and NOE spectral analysis together with electronic circular dichroism (ECD) method.

Zhang’s group isolated two new analogues, cochlioquinones G (**24**) and H (**25**) with cytotoxic effects from the endophytic fungus *B. sorokiniana* A606 collected from the medicinal plant *Pogostemon cablin*. The high-resolution mass spectrometry data revealed a nitrogen atom in the structure of **24**, which was the first report in all cochlioquinones. The NMR spectra, especially the two-dimensional ones, identified an indole-4,7-dione moiety present in this polyketide terpenoid (**24**) [27]. Two highly homologous meroterpenoid biosynthesis gene clusters in *Arthrinium* sp. NF2194 and *Nectria* sp. Z14-w were recently discovered through genomic mining. Media optimization guided by reverse-transcription PCR isolate a series of cochlioquinones including four new ones (arthripenoids D-F, **26**–**28**) and a necripenoid B (**29**), which were determined by NMR spectrometry and single crystal diffraction experiments [28]. In 2019, five new analogues named benzoquinone cochlioquinones I-N (**30**–**35**) were purified from the endophytic fungus *Bipolaris* sp. L1-2 collected from the medicinal plant *Lycium chinense Miller*. Their structures were mainly elucidated through high-resolution mass, NMR spectrometry, chemical transformations, and single-crystal diffraction experiments [29]. 12-keto-cochlioquinone A (**36**) was isolated from the plant pathogenic fungus *B. sorokiniana* 11134. Based on NOE correlation and computational density functional theory (DFT) method together with considering biosynthesis, the structure of new compound (**36**) was determined [30]. In the same year, a new analogue cochlioquinone B derivative (CoB1) (**37**) was found from the endophytic fungus *B. sorokiniana* inhabiting in *Salvia miltiorrhiza Bunge* [31]. Structurally, the polyketide fatty side chain in CoB1 (**37**) was lost, with a methyl group at C-6 and a hydroxyl group at C-11, respectively.

As a co-culture of different microbes together could produce different and new secondary metabolites, which could not be biosynthesized by a single one, two endophytic fungi, *Clonostachys rosea* B5–2 and *N. pseudotrichia* B69–1, were then co-cultivated, and a unique cochlioquinone analogue (**38**), possessing a furan ring, was then obtained [32]. Two possible structures might exist in **38** as the NMR spectra especially HMBC correlations could not differentiate 1,2- or 1,4-quinone type. Based on theoretical NMR calculations, the planar structure of **38** was suggested to possess a 1,2-quinone fragment, and its absolute configuration was determined by ECD calculations [32]. In 2021, Kim et al. [33] utilized bioinformatics methods to analyze the quantitative trait loci of rice attacked by the white-backed plant hopper, from which genes related to the resistance to WBPH were then identified. The main gene could biosynthesize cochlioquinone-9 (CQ-9) (**39**), and its structure was deduced by high-resolution mass spectrometry. CQ-9 (**39**) possess the core skeleton of cochlioquinone A (**2**) but with a methyl anchored at C-15 not at C-14, different from other cochlioquinone analogues [33]. This was the first report about cochlioquinone analogue isolated from plants. A series of cochlioquinones were isolated from the potato endophytic fungus *B. eleusines* last year, in which bipolariterpenes B (**40**) and C (**41**) possessed the same indole-4,7-dione moiety as cochlioquinone G (**24**). The structures of **40** and **41** were elucidated by HR-ESI-MS, NMR DP4^+^ probability analysis and electronic circular dichroism (ECD) [34].

## 3. Phenol-Type Cochlioquinones

**Figure 3:**
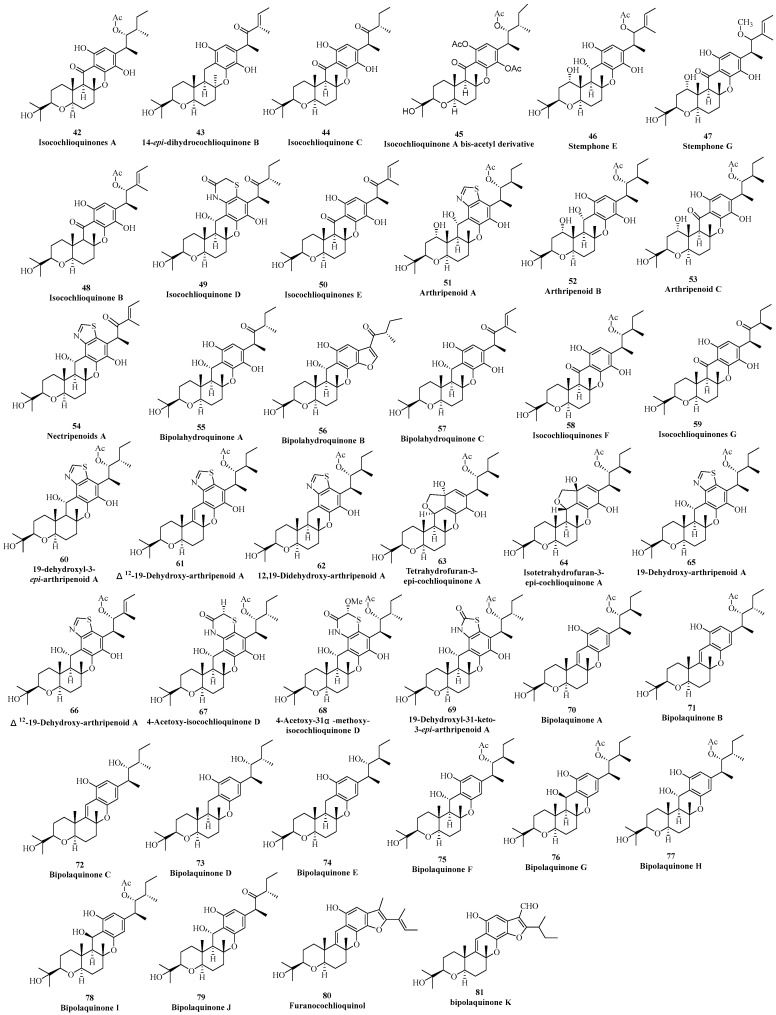
Phenol-type cochlioquinones.

Currently, there are 40 phenol cochlioquinone isolated from different fungi (Figure 3). In 1994, Fukami found the first phenol cochlioquinone isocochlioquinone A (**42**) from the plant pathogenic fungus *B. bieolor* EI-1 [35]. Through NMR spectral analysis and single crystal diffraction experiments, the absolute configuration of the compound (**42**) was determined. In the same year, 14-*epi*-dihydrocochlioquinone B (**43**) was obtained from the fermentation broth of *N. pura*, which was extensively analyzed by one-dimensional and two-dimensional NMR spectral data [15]. According to the plant toxicity tracking method, a new phenolic cochlioquinone isocochlioquinone C (**44**) was isolated from the pathogenic fungus *B. cynodontis cynA*, which exhibited significant plant toxicity and inhibitory activities against mitochondrial electron transfer [18].

An acetylated artificial product isocochlioquinone A bis-acetyl derivative (**45**) was purified from the fungus *D. dematioides* and its structure was mainly characterized by comparison of NMR data with those isocochlioquinone A [36]. In 2008, Tomoda isolated two new analogues stemphones E and G (**46** and **47**) from the fermentation broth of the antagonistic fungus *Aspergillus* sp. FKI-2136 to *Staphylococcus aureus* [23]. The planar structure and absolute configuration of these two new compounds were determined based on ^1^H-NMR, ^13^C-NMR, HSQC, and HMBC, as well as mass spectrometry data. In 2011, Ge’s group isolated isocochlioquinone B (**48**) from the endophytic fungus *Cochliobolus* sp. collected from reed leaves [37]. By analyzing high-resolution mass spectrometry, one-dimensional and two-dimensional NMR, the planar structure and relative configuration of **48** were inferred. Isocochlioquinones D and E (**49** and **50**) with cytotoxic effects were separated from the endophytic fungus *B. sorokiniana* A606 isolated from *Pogostemon cablin* [27]. The high-resolution mass spectrometry data indicated nitrogen and sulfur atoms present in the structure, which was elucidated via NMR spectra to suggest a benzothiazide ring in isocochlioquinone D (**49**).

Two highly homologous meroterpenoid biosynthesis gene clusters in *Arthrinium* sp. NF2194 and *Nectria* sp. Z14-w were analyzed through genomic mining approaches. These gene clusters could biosynthesize cochlioquinones, from which four new ones, arthripenoids A–C (**51**–**53**) and nectripenoid A (**54**) were found, and their structures were determined through high-resolution mass spectrometry, one-dimensional and two-dimensional NMR spectroscopy, and single-crystal X-ray diffraction experiments [28]. Arthripenoid A (**51**) was the first cochlioquinone containing a thiazole ring. Five phenol-type and cytotoxic cochlioquinones bipolahydroquinones A–C and isocochlioquinones F–G (**55**–**59**) were purified from the plant endophytic fungus *Bipolaris* sp. L1-2 inhabiting in *Lycium chinense* Miller [29]. Their structures were determined through a combination of high-resolution mass spectrometry, NMR spectroscopy, chemical transformations, and single-crystal X-ray diffraction experiments. Among them, **56** was the first cochlioquinone with a coumarone moiety. 19-dehydroxyl-3-*epi*- arthripenoid A (**60**) possessing the same thiazole ring as that of arthripenoid A (**51**) was isolated from the plant pathogenic fungus *B. sorokiniana* 11134 in 2020 [31]. In the same year, Zhang’s group mined nine novel phenolic cochlioquinones from the soil-derived fungus *B. zeicola*, namely Δ^12^-19-dehydroxy- arhripenoid A (**61**), 12,19-didehydroxy-arthripenoid A (**62**), tetrahydrofuran-3-*epi*-cochlioquinone A (**63**), isotetrahydrofuran-3-*epi*-cochlioquinone A (**64**), 19-dehydroxy-arthripenoid A (**65**), Δ^2^-19-dehydroxy-arthripenoid A (**66**), 4-acetoxy-isocochlioquinone D (**67**), 4-acetoxy-31α-methoxy-isocochlioquinone D (**68**), and 19-dehydroxyl-31-keto-3-*epi*-arthripenoid A (**69**). Their absolute configurations were determined through comprehensive spectral data, single crystal X-ray diffraction analysis, and ECD calculations. Overall, **61**, **62**, **65** and **66** had a rare thiazole ring, **63** and **64** were the first tetrahydrofuran ring compound with a 6/6/6/6/5 pentacyclic system, **67** and **66** had a rare thiazine moiety, and **69** was the first cochlioquinone with a distinct 2-carbonyl-thiazole ring moiety [38]. The same group the next year re-isolated ten phenolic cochlioquinones bipolaquinones A–J (**70**–**79**) from the same fungus, and their structures were established NMR spectroscopy, X-ray single crystal diffraction, circular dichroism spectroscopy, computational ECD, and hydrolysis reactions [39]. Interestingly, different chirality at C-3/C-12 was found in **73**–**78**, indicating that the methyltransferase and oxygenase had no stereospecificity at C-3 and C-12, respectively, which led to the chemical diversity of cochlioquinones. In 2021, Shiono obtained the cytotoxic compound furano-cochlioquinol (**80**) by co-cultivation of two endophytic fungi *C. rosea* B5–2 and *N. pseudotrichia* B69–1, and different methods including HR-MS, NMR spectroscopy, and calculated ECD to establish its structure [32]. Bipolaquinone K (**81**) with an additional ring was isolated from plant pathogenic fungus *B. sorokiniana* based on UPLC-Q-TOF-MS/MS experimental analysis [40].

## 4. NMR Spectral Features of Cochlioquinones

**Figure 4:**
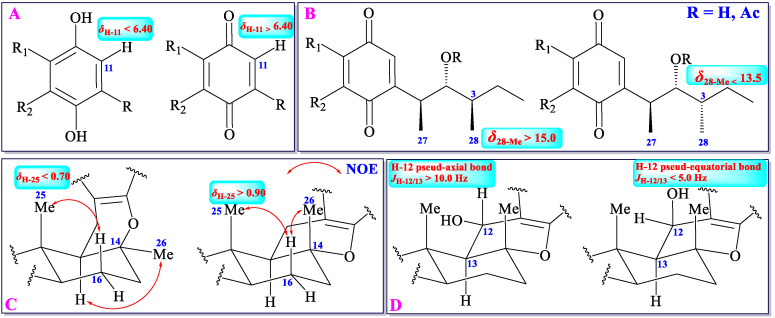
^1^H/^13^C NMR data of different substitutes or configurations.

Usually, chemical shift values of a proton or a carbon is the reflection of electronic cloud density, which is mainly affected by chemical environments around (namely different groups or different configurations). As mentioned above, cochlioquinones can be classified into two types: benzoquinone-type and phenol-type. Analysis of the ^1^H-NMR spectral data in all benzoquinone-type and phenol-type cochlioquinones demonstrates that the ^1^H-NMR chemical shift value of H-11 in benzoquinone-type is over 6.40, whereas the value in phenol-type is lower 6.40 (in CDCl_3_) (Figure 4) [12,13,14,15,16,17,18,19,20,21,22,23,24,25,26,27,28,29,30,31,32,33,34,35,36,37,38,39,40]. Thus, it could judge whether an unknown cochlioquinone is the benzoquinone-type or the phenol-type based on the ^1^H-NMR chemical shift value of H-11.

Methyltransferase can add two methyls (27-Me and 28-Me) to C-3 and C-5 in cochlioquinones, respectively. The configuration of 27-Me is *β*-orientation in all cochlioquinones, whereas the configuration of 28-Me can be *α* or *β*-orientation [12,13,14,15,16,17,18,19,20,21,22,23,24,25,26,27,28,29,30,31,32,33,34,35,36,37,38,39,40]. The different configurations of 28-Me engender different chemical environments, which leads to different chemical shift values of this methyl group. Usually, the ^13^C-NMR chemical shift value of 28-Me is over 15.0 in *β*-orientation, whereas in *α*-orientation, the value is lower 13.5 (in CDCl_3_) (Figure 4) [12,13,14,15,16,17,18,19,20,21,22,23,24,25,26,27,28,29,30,31,32,33,34,35,36,37,38,39,40].

The 26-Me is orientated as a pseud-axial bond in most of cochlioquinones, but the orientation of this methyl in *epi*-cochlioquinone D (**22**) and 12-α-hydroxy-*epi*-cochlioquinone D (**23**) is put as a *α*-orientation (pseud-equatorial bond) [26], which might place 25-Me in the shielding zone of the benzoquinone or the phenol ring, and then the ^1^H-NMR chemical shift value of 25-Me will be in a higher field (*δ*_25-Me_ < 0.70). Thus, according to the ^1^H-NMR chemical shift value of 25-Me, the relative configuration of this group could be established (Figure 4) [12,13,14,15,16,17,18,19,20,21,22,23,24,25,26,27,28,29,30,31,32,33,34,35,36,37,38,39,40].

The H-13 is orientated as a pseud-axial bond in all cochlioquinones, and C-12 could be oxygenated to produce a hydroxyl group with an *α*- or *β*-orientation, which leads to the H-12 to be a pseud-axial bond or pseud-equatorial bond. If the coupling constant of *J*_H-12/H-13_ is over 10.0 Hz, the H-12 is orientated as a pseud-axial bond. If the coupling constant of *J*_H-12/H-13_ is lower 5.0 Hz, the H-12 is orientated as a pseud-equatorial bond. According to the *J*_H-12/H-13_ value, the relative configuration of H-12 could be established (Figure 4) [12,13,14,15,16,17,18,19,20,21,22,23,24,25,26,27,28,29,30,31,32,33,34,35,36,37,38,39,40].

## 5. Mass Spectral Features of Cochlioquinones

The mass fragmentation patterns of cochlioquinones were recently analyzed by Ding’s group [40]. The results revealed that neutral loss (H_2_O, and acetic acid), McLafferty rearrangement, and Retro Diels–Alder (RDA) reactions were found to be the main fragmentation patterns.

**Figure 5:**
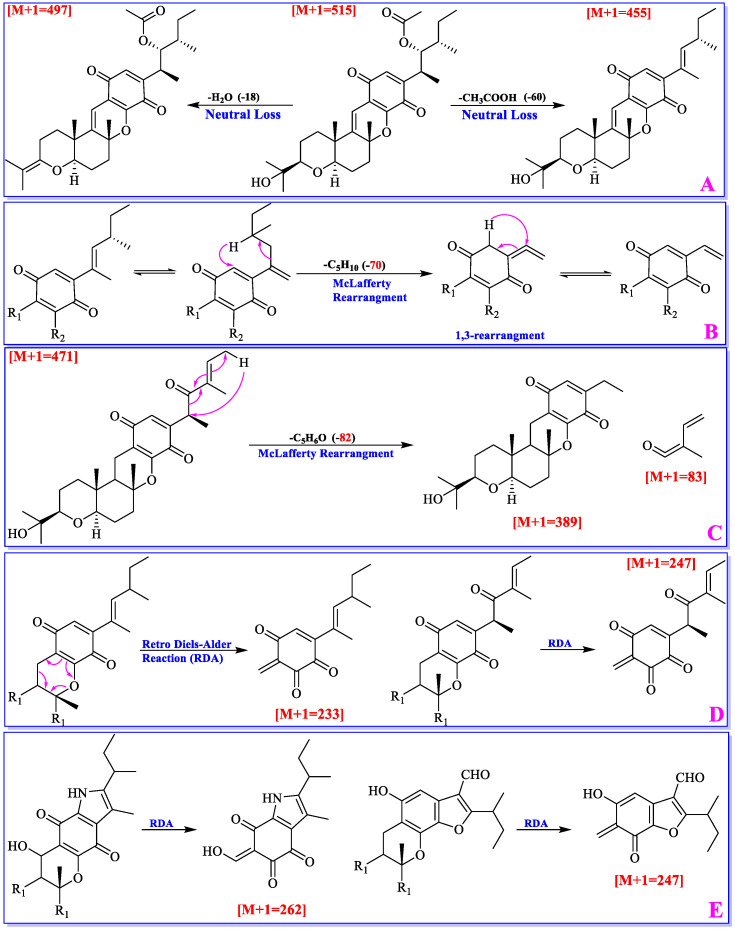
Mass spectral features of cochlioquinones.

If a cochlioquinone possesses a hydroxyl or acetyl group, which easily lost one molecule of H_2_O (-18) or acetic acid (-60) through neutral loss (Figure 5), and the abundance of the parent ion would be relatively low in the mass profile. The Ac group in cochlioquinones is only anchored at C-4. Thus, if the different value from a parent ion to a daughter ion is 60, it implies that an Ac group might be located at C-4. A hydroxyl group can be placed at C-4/C-12/C-19/C-22, respectively, and according to the loss of H_2_O (-18), it can suggest how many hydroxyls are contained in the structure.

McLafferty rearrangement reaction mainly happens on the polyketide part. If C-4/C-5 is a double bond, 1,3-rearrangement will make it to be a C-5/C-27 double bond. Then, a molecule of C_5_H_10_ will be lost through McLafferty rearrangement; If C-4 is a carboxyl group, a molecule of C_5_H_6_O will be lost through McLafferty rearrangement (Figure 5). As a quinone-pyran or a benzo-pyran ring is present in cochlioquinones, Retro Diels-Alder reaction will lead to production two neutral ions (Figure 5). Based on the molecular weight of fragmented ions, different groups on the chains could be suggested.

## 6. The Biological Activities of Cochlioquinones

### 6.1. Plant Toxic Activities

The majority of cochlioquinones are isolated from plant pathogens or endophytic fungi with notable phytotoxic effects. For example, compounds **1–3**, **6–11**, **42**, and **44** were reported to possess phytotoxic activities through the inhibitory effects of rice/corn root growth. Compounds **1–3** and **42** showed certain inhibitory root growth toxicity at concentrations of 20 ppm and 100 ppm, and compound **2** being the most potent, with relative inhibition rates of root growth reaching 9.7% and 8.6% for rice and wheat at a concentration of 100 ppm [35]. At a concentration of 100 ppm, compound **6** exhibited a root growth inhibition rate of 50% against Italian ryegrass, though its inhibition rate was significantly lower than that of compounds **2** and **3** [41]. Uneo also discovered that compounds **2**, **3**, and **6**–**11** exhibited inhibitory growth activity against the roots of both Italian ryegrass and rice at a concentration of 100 ppm [18]. At this concentration, compounds **2**, **7**, **9**, **42**, and **44** exhibited absolute inhibitory activity, while compounds **6**, **9**, and **10** had the weakest activity. This might be attributed to the substitution of a carbonyl group in the benzoquinone ring, leading to a decrease in activity. Further mechanistic studies revealed that compounds **2**, **3**, and **6**–**11** exhibited competitive inhibitory effects on NADH oxidoreductase in the mitochondrial electron transfer system, which might be one of the factors of plant disease [18]. Furthermore, this inhibitory effect was related to the hydrophobicity of the compounds, and it was just one of the decisive factors. The interactions between inhibitors and binding sites might also be influenced by spatial and electronic properties, necessitating further research to elucidate these mechanisms [18].

### 6.2. Antiparasitic and Insecticidal Activities

In the study of the interaction between such fungal toxins and plants, it has also been found that some compounds exhibit certain insect-resistant and anti-parasitic properties. However, there is limited research in this area, and to date, only five cochlioquinones with such effects have been identified. Experiments demonstrated that compound **2** possessed nematocidal activity with an ED_50_ of 135 μM in 1990 [42]. Additionally, it was revealed that compound **2** was a competitive inhibitor of ivermectin binding to the cell membrane of *C. elegans*, with an inhibition constant Ki of 30 μM, and it may share a similar mode of action with ivermectin. Then, it was found that compounds **3** and **42** significantly inhibited the growth of *Plasmodium falciparum* ([IC_50_] < 5.1 μg/mL) [43]. In 2021, Kim et al. utilized bioinformatics methods to analyze the quantitative trait locus (QTL) in rice after it was invaded by the white-backed planthopper (WBPH), and they identified a gene related to resistance to WBPH. [33,44] They localized the main gene for compound **39** synthesis and isolated it from the green leaves of resistant plants. It was observed that after treatment with compound **39**, the growth of rice plants was better than that without treatment. In addition, the expression of plant defense genes increased, which suggested that it can be used as a new environmentally friendly insecticide to control the damage caused by WBPH to rice.

### 6.3. Antibacterial Activities

With the emergence of drug resistance in bacteria and fungi, the use of antibiotics has become increasingly limited. The exploration of novel antibacterial drugs from natural products has emerged as a major direction. According to current research, most cochlioquinones show antibacterial activity. When compound **1** was isolated in 1968, it was found to inhibit the growth of *Bacillus megaterium* and *Sarcina lutea* at a dose of 10 μg [12]. Then, Hansske et al. reported weak antibacterial activity of compounds **4** and **43** against several strains, including *Acinetobacter calcoaceticus*, *Arthrobacter citreus*, and *Bacillus brevis* [15]. However, compound **2** showed inhibitory activity against *Bacillus subtilis*, while compound **43** did not. Additionally, the fungus *Epicoccum purparascens* was more sensitive to compound **43**, while *Mucor miehei*, *Phytophthora infestans*, and *Venturia cerasi* were more sensitive to compound **2**. These findings suggested that there was no specificity in the antibacterial activity. Wright et al. reported moderate antifungal activity of compounds **3**, **42**, and **44** against *Microbotryum violaceum* in 2002 [43]. Subsequently, compounds **2** and **45** were found to exhibit significant differences in their inhibitory effects on *Aspergillus niger*, *S. aureus*, and *B. subtilis* [36]. The minimum inhibitory concentration (MIC) of compound **45** was approximately nine times that of compound **2**, suggesting that the quinone moiety played a significant role in antibacterial activity.

Omura et al. assessed those compounds **8**, **15**, and **16** showed no inhibitory effect on methicillin-resistant *S. aureus* (MRSA), but they could enhance the antibacterial activity of imipenem against MRSA, with compound **16** being the most potent and compound **8** being the least potent [21]. This enhancement may be attributed to the acetyl group at C-4. Additionally, they investigated the antibacterial enhancement effects of compound **16** on other antimicrobial drugs against MRSA and found that it significantly enhanced the antibacterial activity of imipenem, cloxacillin, cefazolin, and other *β*-lactam antibiotics against MRSA. In 2008, the research group investigated the structure–activity relationship of cochlioquinones by evaluating the antibacterial activities of compounds **15**, **16**, **18**, **19**, **46**, and **47** and **21** chemical derivatives [23]. They found that derivatives with an acetyl group at C-4 exhibited enhanced activity, but longer acyl residues at C-4 led to a loss of this activity. It would suggest that moderate lipophilicity at C-4 was important for maintaining activity. For position C-11, the introduction of most *O*-alkyl residues led to a decrease in activity, with only hydroxyl and *O*-methyl groups showing weak activity. Therefore, modification of this position may enhance activity [23]. Moreover, when methyl or acetyl residues were introduced into the C-10 hydroxyl group, activity was lost. For positions C-12 and C-19, enhanced activity remained consistent, regardless of the introduction of hydroxyl groups. However, the mechanism of enhancement was not clarified. Among these compounds, compound **46** exhibited the most significant increase in the activity of imipenem against MRSA (533 times) while maintaining the lowest cytotoxic activity (human peripheral blood leukemia T cells and human peripheral blood leukemia T cells) [45].

Wang et al. reported that compound **20** inhibited MRSA, *Escherichia coli*, *Pseudomonas aeruginosa*, *Pseudomonas fluorescens*, and *S. epidermidis* with MIC values of 8, 4, 4, 0.5, and 0.5 mg/mL, respectively, in 2012 [24]. Notably, the activity against *E. coli*, *P. fluorescens*, and *S. epidermidis* was more potent than that of the positive control (ampicillin, with the MIC values being 8, 4, and 4 mg/mL, respectively). Compounds **22** and **23** were found to inhibit the growth of *C. miyabeanus* [26]. Notably, *C. miyabeanus* was found to biosynthesize cochlioquinones, with reverse chirality detected at the C-14 position. Besides, it also exhibited weaker inhibitory activity against itself. It was confirmed that compounds **2**, **3**, **7**, **8**, **31–35**, **42**, **44**, **58**, and **59** had inhibitory effect on *B. subtilis*, *Clostridium perfringens*, *E. coli*, and *P. aeruginosa* in 2019 [29]. The author observed that compounds **3**, **7**, and **44** exhibited antibacterial activity with an MIC value of 26 μM. The observed activity may result from their shared polyketide side chains and the lack of substitution at C-11. One year later, Feng et al. reported that compounds **2**, **3**, **8**, **29**, **44**, and **48** exhibited inhibitory effects against *S. aureus*, MRSA, and four clinical strains of *S. aureus* (8–21, 6281, 309-1, and 309-6), with the MIC values ranging from 12.5 to 100 μg/mL [30]. Among them, compound **3** exhibited the strongest activity, with the MIC value being 12.5–25 μg/mL. Compound **44** showed moderate inhibitory effects against two clinical strains of *S. aureus* (8–21 and 309-6), with an MIC value of 25 μg/mL. Compound **2** had an MIC value of 12.5 μg/mL against *S. aureus* and MRSA but showed no activity against the four clinical strains at a concentration of 100 μg/mL. Additionally, compound **3** demonstrated weak antifungal activity against *Candida albicans*, with an MIC value of 100 μg/mL. However, after 25% ketoconazole was added to the *C. albicans* suspension, compound **3** exhibited synergistic inhibitory activity against *C. albicans*, with an MIC value of 12.5 μg/mL [30]. In the same year, studies demonstrated that compound **37** effectively inhibited infections mediated by *P. aeruginosa* [31]. Based on *P. aeruginosa* infection model, mice treated with it showed reduced lung injury, reduced systemic spread of bacteria, lower mortality rates, and reduced inflammatory responses compared to wild-type mice. The results indicated that compound **37** enhanced alveolar macrophage survival and bacterial clearance by suppressing PAK1/Akt/mTOR-mediated protective autophagy. Thereby, it provides a molecular basis for regulating the host immune response to *P. aeruginosa* infections and indicates that it is a potential treatment option for infectious diseases [31]. Zhou et al. reported that compound **16** showed antibacterial activity against *Erysipelothrix rhusiopathiae* WH13013 and *Streptococcus suis* SC19 in 2003 [46]. The MIC values for these two pathogens were found to be 1.56 and 6.25 μg/mL, respectively. Furthermore, the antibacterial activity of it was found to be superior to that of penicillin, with the MIC value being 6.25 μg/mL.

### 6.4. Cytotoxic Activities

Based on the current literature, cochlioquinones have demonstrated significant cytotoxic effects on various tumor cells, indicating their potential for therapeutic applications. Lawrence et al. reported the toxicity of compound **1** in 10-day-old chicken embryos in 1968 [12]. When injected into the yolk sac at a dosage of 100 pg, the mortality rate within 24 h was 33%, significantly higher than the 5% observed in the control group. Additionally, compound **1** caused mortality in zebrafish larvae, with an average LC_50_ of 1.6 μg/mL within 24 h [12]. Hansske et al. documented the cytotoxic effects of compounds **4** and **43** in 1994 [15]. Specifically, compound **4** inhibited the growth of mouse embryo fibroblasts (Balb 3T3) at a concentration of 5 μg/mL and induced cell lysis at 25 μg/mL. Both compounds **4** and **43** caused morphological changes in young hamster kidney (BHK) cells at 6.5 μg/mL and completely lysed the BHK cell line at 12.5 μg/mL. However, at concentrations exceeding 20 μg/mL, only weak growth inhibitory activity was observed against mouse leukemia cells (L1210) [15].

Compounds **17** and **44** were reported to exhibit cytotoxic effects against human cervical cancer cells (HeLa), with IC_50_ values of 6.5 and 5.9 μg/mL, respectively, in 2007 [22]. Tomado et al. found that among compounds **8**, **15**, **16**, **18**, **19**, **44**, and **45**, compound **8** displayed the strongest cytotoxic activity against Jurkat cells, with an IC_50_ value of 0.07 μg/mL in 2008 [23]. Compound **20** was reported to exhibit potent cytotoxic effects on human breast cancer cells (MCF-7), human pancreatic cancer cells (SW1990), and human liver cancer cells (SMMC7721), with IC_50_ values of 4, 5, and 7 mg/mL, respectively in 2012 [24]. It also demonstrated moderate cytotoxic activity against human liver cancer cells (HepG2), human large cell lung cancer cells (NCI-H460), and human prostate cancer cells (DU145), with IC_50_ values of 20, 11, and 17 mg/mL, respectively. Notably, the cytotoxic activity of compound **20** against the SW1990 cell line was stronger than that of the positive control fluorouracil, which had an IC_50_ value of 16 mg/mL [24].

Compounds **17**, **21**, **42**, and **44** were found to induce apoptosis in the HCT116 cell line in 2014 [25]. Among these, compound **17** was the most toxic, with an effective concentration range of 10 to 30 μM, inducing apoptosis in a dose-dependent manner at doses of 10, 20, and 30 μM. Treatment with compound **17** resulted in the down-regulation of Bcl-2 expression in HCT116 cells, while Bcl-xL expression remained unchanged. Bcl-2 is an important anti-apoptotic protein, and Bcl-xL, as a pro-survival protein, prevents the release of mitochondrial contents such as cytochrome C [47]. Therefore, compound **17** may exert its cytotoxic activity by inducing mitochondrial-mediated apoptosis in the HCT116 cell line.

Hashimoto et al. reported that compounds **2**, **5**, **7**, **8**, **22**, and **23** exhibited strong cytotoxic activity against human colorectal adenocarcinoma cells (COLO 201) in 2015 [26]. This suggested that the cytotoxic activity was not associated with the chirality at position C-14 or with the polyketide side chain substitution. Compounds **42** and **44** showed weaker activity, possibly due to their non-reactive phenolic-type structures, although the exact reasons remain unclear. Compounds **3**, **7–9**, **24**, **25**, **44**, **47**, and **48** were reported to exhibit significant cytotoxic effects on four human tumor cell lines: SF-268, MCF-7, NCI-H460, and HepG-2 in 2016 [27]. Among these, compounds **3** and **7–9**, which possess quinone structural characteristics, exhibited broad inhibitory effects on various cell lines, with activity comparable to cisplatin. Compound **8** showed the strongest inhibitory activity against SF-268 and HepG-2 cell lines, with IC_50_ values of 1.5 μM and 1.2 μM, respectively. The selectivity of compound **8** towards the NCI-H460 cell line may be associated with the dehydrogenation at C2/C3 to form a double bond. The selectivity of compounds **7** and **3** towards the NCI-H460 cell line may be related to the hydroxyl group at C-12, which increases toxicity in the absence of a hydroxyl group [27]. In contrast, the introduction of hydroxyl or other groups at C-11 significantly reduced the toxicity of the compounds. Compounds **50** and **44** exhibited stronger selective inhibitory toxicity towards SF-268 and HepG-2 cell lines, with IC_50_ values of 17.2 and 13.6 μM, respectively. The analysis indicated that the presence of dehydrogenation between C-2 and C-3 and a hydroxyl group at C-10 resulted in stronger selective cytotoxic activity towards SF-268 and HepG-2 cell lines [27].

Gao et al. tested the cytotoxicity of compounds **2**, **3**, **7**, **8**, **30**–**35**, **42**, **44**, and **55**–**59** against NCI-H226 and MDA-MB-231 cell lines in 2019 [29]. The results showed that compounds **8** and **57** exhibited considerable cytotoxicity, with IC_50_ values of 5.5, 6.9, 6.7, and 7.1 μM, respectively (doxorubicin was used as a positive control, with an IC_50_ value of 0.3 μM). Compounds **30**–**33** exhibited selective cytotoxic activity against the MDA-MB-231 cell line, with IC_50_ values of 8.5, 9.5, 7.5, and 5.6 μM, respectively [29]. Other compounds showed an inhibition rate below 50% at a concentration of 10 μM. A comparison of these 15 compounds revealed that those with C-2/C-3 double bonds or a methoxy substitution at C-11 and a hydroxyl group at C-12 exhibited cytotoxic activity against the MDA-MB-231 cell line. However, the structural characteristics crucial for cytotoxicity against the NCI-H226 cell line remain undefined [29].

Compounds **61**–**69** were reported to exhibit cytotoxic activities against SW1990, HepG2, MCF7, and HCT116 cell lines as determined by the MTT method in 2020 [38]. The results showed that compounds **61** and **62** exhibited selective cytotoxic activity against the SW1990 cell line, with IC_50_ values of 21.6 and 25.9 μM, respectively. Compound **64** exhibited moderate cytotoxicity against HepG2, MCF7, and HCT116 cell lines, with IC_50_ values ranging from 18.4 to 29.4 μM [38]. Compounds **8**, **29**, **38**, and **80** were reported to exhibit cytotoxic activities against human promyelocytic acute leukemia cells (HL60), with IC_50_ values of 1.61, 0.93, 0.63, and 0.47 μM, respectively (camptothecin served as a positive control, with an IC_50_ value of 0.016 μM) in 2021 [32]. The inhibitory activity of compounds **8** and **29** suggested that the C-12/C-13 double bond is a key active group for cellular toxicity. A comparative analysis of the cytotoxicity of compounds **8**, **38**, and **80** indicated that the introduction of a furan ring increased the cellular toxicity of these compounds. Compared to compounds **38** and **80**, the phenolic skeleton exhibited higher cytotoxic activities than the benzoquinone form [32].

Zhou et al. reported that compound **16** exhibited cytotoxic effects against various cancer cells, including human prostate cancer cells (22Rv1 and PC-3), HepG2, human non-small cell lung cancer cells (A549), HeLa, human normal prostate stromal immortalized cells (WPMY-1), and mouse embryonic osteoblast precursor cells (MC3T3-E1) in 2023 [46]. The strongest cytotoxicity was observed against the PC-3 cell line, with an IC_50_ value of 2.77 μM. Additionally, compound **4** reduced PC-3 cell line colony formation in a dose-dependent manner, induced apoptosis, and blocked the cell cycle in the S phase. It may be a potential anti-proliferative agent and a promising lead compound for the treatment of prostate cancer [46].

In addition to inducing apoptosis and inhibiting cell proliferation, cochlioquinones also exhibit cytotoxic effects by inhibiting key enzymes that regulate cell proliferation. In 1995, Ogawara et al. reported that compound **2** exhibited specific inhibitory activity against diacylglycerol kinase [48]. Kinetic experiments indicated that compound **2** inhibited neither protein kinase C nor epidermal growth factor receptor-related protein tyrosine kinase, making it a specific inhibitor of diacylglycerol kinase, which regulates the activity of protein kinase C. Protein kinase C, in turn, regulates the process of cell proliferation [52]. Diacylglycerol kinase α (type I isomer) was reported in 2007 to inhibit apoptosis in human melanoma cells induced by tumor necrosis factor alpha (TNF-α) by activating nuclear factor kappa B (NF-κB) [53]. Therefore, compound **2** can inhibit NF-κB and the phosphorylation of protein kinase C by inhibiting diacylglycerol kinase, thereby promoting the apoptosis of cancer cells.

In 2004, it was reported that compounds **2**, **5**, **13**, **14**, and **42** effectively competed with macrophage inflammatory protein 1α (MIP-1α) for binding to human chemotactic factor receptor CCR5, with IC_50_ values of 11, 4, 100, 7, and 50 μM, respectively [20]. The human chemotactic factor receptor acts on G protein-coupled receptors and is one of the main co-receptors for human immunodeficiency virus type 1 (HIV-1) to invade host cells. Therefore, the binding of compounds **2**, **5**, **13**, **14**, and **42** to receptor CCR5 may have the potential to prevent HIV-1 from entering cells. A comparative analysis of compounds **2** and **42** revealed an increase in the activity of the benzoquinone skeleton, while compound **13** showed a significant decrease in activity after methoxy substitution at C-11 [20]. Compounds **5** and **14** had similar activity to compound **2**, despite having different stereochemical properties at C-14, indicating that the chirality at C-14 was not the functional group responsible for the activity [20].

Compound **81** showed strong cytotoxic activities against the B16 cell line, with IC_50_ values of 1.91 μM, suggesting that the dehydrogenation between C-12 and C-13 might contribute to this activity in 2024 [40].

### 6.5. Immunosuppressive Activities

During the screening process of cell activity, it was found that such cochlioquinones not only have cytotoxicity against various tumor cells but also have certain activity against human immune cells. In 2018, Ge et al. evaluated the immunosuppressive activity of compounds **26**–**29** and **49**–**52**, and they found that compound **51** had inhibitory effects on ConA-induced T cell proliferation [28]. Specifically, activated T cells secreted a large amount of TNF-α and interferon-gamma (IFN-γ) under ConA stimulation. After the treatment with compound **51**, the content significantly decreased, with the IC_50_ value being 4.2 mM. Therefore, compound **51** had immunosuppressive activity against CD4 + T cells. Compounds **59**–**67** were reported to perform immunosuppressive activity on ConA-induced T lymphocyte proliferation in vitro in 2021 [38]. The results showed that compounds **59**, **60**, **63**, **64**, and **67** all had a thiazole ring structure and exhibited significant inhibitory activity against ConA-induced T lymphocyte proliferation, with the IC_50_ values ranging from 5.6 to 8.8 μM. However, compounds **65** and **66** had a 2H-1, 4-thiazine ring structure and did not exhibit inhibitory activity. These results suggested that thiazole functional groups may play an important role in their activity. These active compounds may serve as lead compounds for the treatment of immune-related diseases and be used to design and develop new immunosuppressive drugs. Zhang’s group evaluated the immunosuppressive activity of compounds **56** and **68**–**77** on ConA-induced T lymphocyte proliferation, and they found that compounds **69**–**70** and **74**–**77** had significant inhibitory activity, with the IC_50_ values ranging from 4.1 to 9.4 μM in 2021 [39]. This also provided potential guiding molecules for the design and development of new immunosuppressive drugs for the treatment of autoimmune-related diseases.

### 6.6. Related to Lipid Metabolism

Compound **5** was identified as a potent inhibitor of acyl-CoA: cholesterol acyltransferase (ACAT), exhibiting an IC_50_ value of 1.7 μM [16]. This compound demonstrated a selective inhibitory effect, as its activity against plasma lecithin: cholesterol acyltransferase (LCAT) was significantly weaker, with an IC_50_ value of 15.8 μM, approximately 10-fold higher than that for ACAT. The selective inhibition of ACAT by compound **5** is of particular interest, as ACAT plays a critical role in cholesterol esterification, a key step in intestinal cholesterol absorption and hepatic secretion of very low-density lipoprotein (VLDL) cholesterol. By inhibiting ACAT, compound **5** has the potential to reduce serum cholesterol levels, thereby mitigating lipid accumulation in arterial walls and promoting the regression of atherosclerotic lesions. In vivo studies further supported these findings, demonstrating that compound **5** significantly inhibited cholesterol absorption in cholesterol-fed rats. At a dose of 75 mg/kg, cholesterol absorption was reduced by 50%, highlighting its potential as a therapeutic agent for hypercholesterolemia and related cardiovascular diseases [16]. Compounds **2** and **12** were reported to inhibit diacylglycerol acyltransferase (DGAT), with IC_50_ values of 6.3 and 5.6 μg/mL, respectively (using Evocarpine as a positive control, IC_50_ = 6.8 μg/mL) [45]. DGAT is a key enzyme in the triacylglycerol (TAG) biosynthesis pathway, catalyzing the final and rate-limiting step in the conversion of diacylglycerol (DAG) to triacylglycerol. Given its central role in lipid metabolism, DGAT has emerged as a promising therapeutic target for the treatment of metabolic disorders such as obesity, hypertriglyceridemia, and type 2 diabetes. The inhibitory activity of compounds **2** and **12** against DGAT suggests their potential as lead compounds for the development of novel DGAT inhibitors. Such inhibitors could offer therapeutic benefits by modulating lipid metabolism, reducing fat accumulation, and improving insulin sensitivity, thereby addressing the underlying metabolic dysregulation associated with obesity and related conditions [45].

### 6.7. Related to Angiogenesis and Platelet Aggregation

In 1994, Hansske et al. reported that compound **4** exhibited significant inhibitory effects on human and bovine platelet aggregation induced by various stimuli [15]. In contrast, compound **43** showed no such inhibitory activity, which may be attributed to the absence of a benzoquinone skeleton, a structural motif hypothesized to be essential for the antiplatelet activity. Further studies revealed that compound **12** demonstrated potent anti-angiogenic properties by inhibiting the proliferation of bovine aortic endothelial cells (BAECs) at a concentration of 1 mg/mL, without exhibiting cytotoxicity [19]. Notably, Compound **12** exhibited stronger inhibitory activity against BAECs compared to both normal and tumor cell lines, indicating its selectivity for endothelial cells. More recently, Kim et al. investigated the pharmacological effects of compound **39** in a murine model of sepsis induced by cecal ligation and puncture (CLP) [31]. Compound **39** was found to increase the survival rate of CLP mice by 60%, demonstrating its potential as a therapeutic agent for sepsis. Additionally, compound **39** exhibited significant vasodilatory effects on rat aortic rings and markedly reduced collagen-induced platelet aggregation in rats.

## 7. Biosynthesis of Cochlioquinones

Isotope-labelling experiments, bioinformatic analysis for gene clusters and enzyme catalysis confirmed that cochlioquinones as a member of polyketide-terpenoid hybrid molecules (PK-TPs) were biosynthesized via polyketide synthase, prenyl-transferase, terpenoid cyclase and other tailoring enzymes [28,51,52,53].

**Figure 6:**
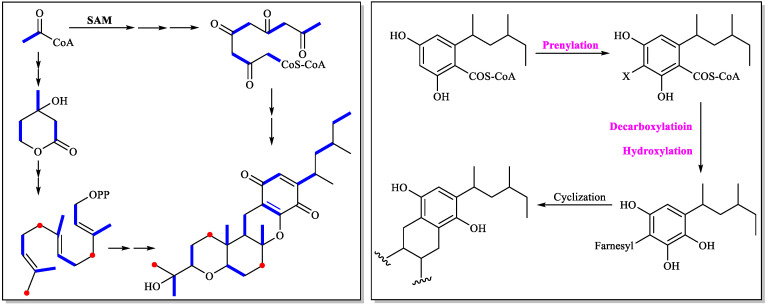
Cochlioquinone biosynthesis investigated by isotope-labelling experiments.

Scolastico et al. first investigated the cochlioquinone biosynthesis, and confirmed that the C_15_ terpenoid unit (A, B, and C rings) in the cochiloquinones A and B incorporated [2-^14^C] mevalonolactone, and the methyl groups in the polyketide part were originated from [Me-^14^C] methionine through isotope-labelling experiments [49]. Later, the same group used ^18^O_2_ isotope-labelling experiments combined with chemical degradation approaches to reveal that the two oxygen atoms of the 2-(2-hydroxyl-propyl) tetrahydropyran system of cochiloquinones A and B were derived from two different molecules of oxygen [50]. The biosynthetic sequence of cochiloquinones was also analyzed by isotopic tracer experiments with use of ^13^C (acetate), ^14^C (MeI) and ^18^O_2_. The possible biosynthetic pathway was then suggested: prenylation of the acetogenin-derived aromatic nucleus, decarboxylation and hydroxylation in the same carbon, and finally cyclization of the terpenoid unit to form the end product cochiloquinone skeleton (Figure 6) [51].

**Figure 7:**
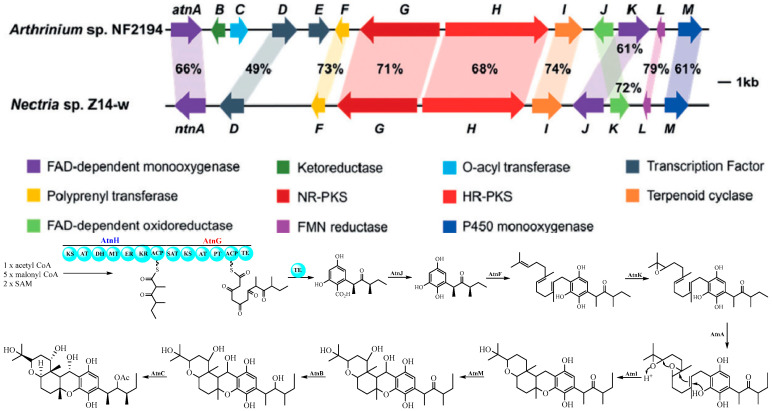
Cochlioquinone biosynthesis investigated by bioinformatic and biochemical analysis [28].

Two polyketide-terpenoid hybrid molecules (PK-TPs) gene clusters encoding polyketide synthase, prenyl-transferase, terpenoid cyclase and other tailoring enzymes (potent cochiloquinone biosynthetic gene cluster) were found based on bioinformatic analysis when Ge’s group analyzed the genomes of two phylogenetically distinct fungi *Arthrinium* sp. NF2194 and *Nectria* sp. Z14-w. Both these fungi could produce cochiloquinones through media optimization and reverse-transcription PCR analysis, though cochiloquinones from *Nectria* sp. Z14-w could not possess acetyl group on the polyketide part, which was completely consistent with comparable gene cluster analysis by absence of the *O*-acyl transferase in the cochiloquinones gene cluster of *Nectria* sp. Z14-w [28]. Then the specific function of each enzyme in the gene cluster of cochiloquinones was characterized by gene deletion, heterologous expression and enzymatic catalysis in vitro. For example, AtnH was responsible for synthesis of the polyketide parts due to the absence of cochiloquinones through by knock-out study, which was also supported by heterologous expression of AtnH/AtnG to produce two polyketide compounds. AtnF was suggested to be a membrane-bound prenyl-transferase by introducing a farnesyl group to the polyketide intermediate, and cochiloquinones were then absent after the genetic inactivation of *atnF*. The gene *AtnI* was suggested to encode a terpenoid cyclase, which might initiate the sequential tricyclic ring (6/6/6-tricyclic ring) formation through protonation of the terminal expoxide. The products as polyketides combined with a farnesyl group from Δ*atnI* mutant supported the above-mentioned conclusion. In addition, the authors also investigated the functions of the P450 oxygenase, and *O*-acyl transferase through enzymatic catalysis in vitro. Many cochiloquinones analogues are postulated to be artificial products through spontaneous oxidation, tautomerization, and rearrangements (Figure 7) [28].

## 8. Discussion

**Figure 8:**
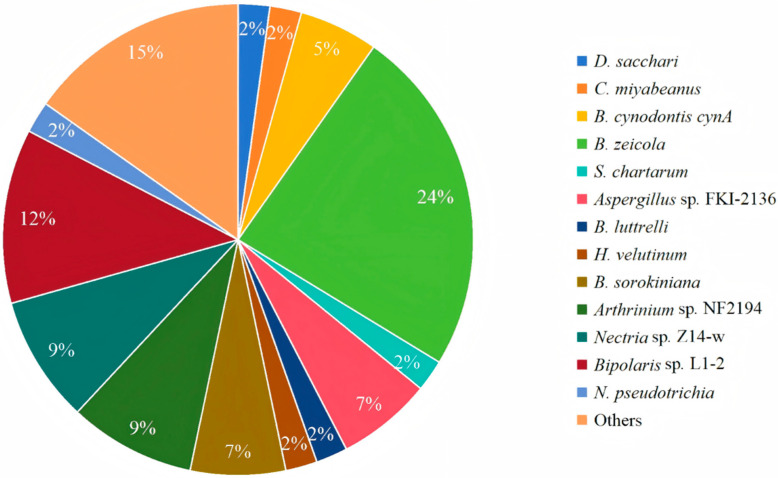
Distribution of producing strains of cochlioquinones.

To date, there are more than 80 cochlioquinones isolated from different resources (Figure 8). Cochlioquinones are characterized by a core 6/6/6/6 tetracyclic ring system, which arises from the fusion of sesquiterpenoid and polyketide moieties. Based on the polyketide part, these compounds are classified into two major groups: benzoquinone-type and phenol-type cochlioquinones. The structural variations among these compounds are primarily due to post-modifications such as esterification, oxidation, heterocyclization, and rearrangement. These modifications not only contribute to structural diversity but also influence their biological activities. For instance, the presence of a quinone moiety in benzoquinone-type cochlioquinones is often associated with enhanced bioactivity, such as cytotoxicity and antibacterial effects, compared to their phenol-type counterparts.

Key NMR features, such as the chemical shift of H-11 and the configuration of methyl groups (e.g., 27-Me and 28-Me), serve as diagnostic markers for distinguishing between benzoquinone-type and phenol-type cochlioquinones. Mass spectrometry further aids in structural characterization by revealing fragmentation patterns, including neutral losses (e.g., H_2_O, acetic acid), McLafferty rearrangements, and retro Diels–Alder reactions.

**Figure 9:**
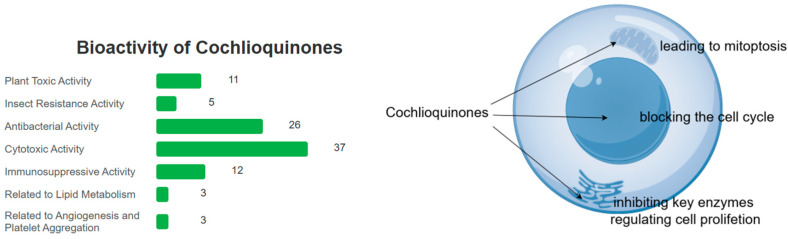
Cochlioquinones mainly lead to apoptosis by blocking the cell cycle, and inhibit key enzymes regulating cell proliferation and mitoptosis.

The major biological activities of cochlioquinones include cytotoxic, antibacterial, antifungal, insecticidal, and plant growth-regulating effects (Figure 9). While some structure-activity relationships (SARs) have been elucidated, the precise mechanisms underlying their inhibition of bacteria, fungi, and insects remain poorly understood and warrant further investigation.

A single fungal species can produce different secondary metabolites with diverse carbon skeletons or ring systems, which leads to varying bioactivities, and different species can also produce same metabolites or analogues with similar bioactivities. This indicates that the biological activities are primarily determined by chemical structure rather than the fungal species. Thus, the biological activities of cochlioquinones are not dependent on the fungal species, but are more closely associated with molecular structural features. For instance, quinone-type compounds generally exhibit stronger cytotoxic and antibacterial activities, while phenolic-type compounds show greater potential in immunomodulation.

The biosynthesis of cochlioquinones involves a hybrid polyketide-terpenoid pathway, orchestrated by polyketide synthases, prenyl-transferases, and terpenoid cyclases. Isotopic labeling and genomic studies have elucidated key steps, including prenylation of the polyketide backbone and subsequent cyclization. Recent advances in bioinformatics and heterologous expression have further clarified the roles of specific enzymes, such as AtnH and AtnF, in the biosynthetic cascade. These insights pave the way for engineered production of novel cochlioquinone derivatives with enhanced bioactivities.

Although significant progress has been made in the structural identification and biological activity screening of cochlioquinones, research on structure–activity relationships (SAR) remains insufficiently systematic, and the contributions of key pharmacophores and stereochemistry are still not well defined. Furthermore, the current toxicological profile of cochlioquinones remains inadequately characterized, with a notable scarcity of data on in vivo safety, chronic exposure risks, and potential off-target effects. This represents a significant gap that must be addressed before any serious consideration for agricultural or therapeutic development. Regarding SAR, future efforts should prioritize integrating computational modeling and target engagement studies to move beyond descriptive correlations and establish causal links between specific structural motifs—such as the quinone moiety, C-2/C-3 unsaturation, and C-12 oxygenation—and their mechanistic outcomes. A focused approach on these high-impact structural features will accelerate the rational design of derivatives with optimized efficacy and reduced toxicity. Studies on mechanisms of action largely remain at the level of phenotypic observation, lacking precise identification of molecular targets. Exploration into the engineering applications of biosynthetic pathways is inadequate, limiting the creation of structural diversity. Furthermore, ecological functions, in vivo efficacy, and pharmacokinetic properties remain underinvestigated areas. Future research should integrate chemical biology and synthetic biology strategies to deeply explore target mechanisms, rational design, and in vivo evaluation.

This review systematically summarizes the chemistry and biology of 81 naturally occurring cochlioquinones, highlighting their structural diversity, NMR and MS spectral features, bioactivities, and biosynthetic pathways. The findings underscore the potential of cochlioquinones as valuable candidates for agricultural and pharmaceutical applications.

## 9. Conclusions and Outlooks

Cochlioquinones, as a class of polyketide-terpenoid hybrid metabolites produced by fungi, possess a distinctive 6/6/6/6 tetracyclic ring system. To date, 81 structurally diverse analogues have been isolated from various plant pathogenic and endophytic fungi. Based on the structural features of the polyketide moiety, they can be classified into two major categories: benzoquinone-type and phenol-type cochlioquinones. These compounds exhibit a broad spectrum of biological activities, including phytotoxicity, antibacterial, cytotoxic, immunosuppressive, and lipid metabolism-regulating effects, demonstrating potential applications in both agricultural and pharmaceutical fields.

Looking forward, several promising research directions merit further exploration. First, the application of advanced machine learning and AI-based predictive models could revolutionize the discovery of novel cochlioquinones by enabling in silico screening of fungal genomes for cryptic biosynthetic gene clusters. Second, the development of efficient heterologous expression platforms in tractable fungal hosts would facilitate the production of rare analogues and engineered derivatives with enhanced bioactivities. Third, integrating chemical biology approaches—such as activity-based protein profiling and CRISPR-Cas9-based gene editing—could elucidate precise molecular targets and mechanisms of action, particularly for immunosuppressive and anticancer activities. Furthermore, structural diversification through combinatorial biosynthesis and semisynthetic modification may yield optimized candidates with improved pharmacological properties. Lastly, advancing ecological studies to understand the natural roles of cochlioquinones in fungal–plant interactions could inspire new strategies for sustainable agriculture, such as developing biocontrol agents or resistance inducers. These interdisciplinary efforts will not only deepen our understanding of cochlioquinone biology but also accelerate their translation into practical applications.

In conclusion, cochlioquinones represent a class of structurally unique and biologically diverse natural products with significant potential as lead compounds for novel agrochemicals and therapeutics. Future research should focus on in-depth mechanistic studies, systematic SAR analyses, and precise regulation and engineering of biosynthetic pathways to facilitate the practical application of these compounds.

## Figures and Tables

**Table 1:** Cochlioquinones isolated from natural sources. Compounds **1**–**41** are classified as benzoquinone-type cochlioquinones, while compounds **42**–**81** are categorized as phenol-type cochlioquinones.

No.	Name	Molecular Formula	Biological Activities	Species	Refs.	Analytical Tools
**1**	Stemphone A	C_30_H_42_O_8_	Cytotoxic effects; antibacterial activity; DGK inhibition; plant toxic activity	*S. sarcinaeforme*; *Mollisia* sp. SCSIO41409; *D. sacchari*	[10,12,14,35]	NMR and X-ray
**2**	Cochlioquinone A	C_3_0H_44_O_8_	DGK inhibition; plant toxic activity; antiparasitic activity; antibacterial activity; cytotoxic activity	*C. miyabeanus*; *D. sacchari*	[13,14,18,20,26,29,30,35,36,42,45,48]	NMR and X-ray
**3**	Cochlioquinone B	C_28_H_40_O_6_	Plant toxic activity; antiparasitic activity; antibacterial activity; cytotoxic activity	*C. miyabeanus*	[13,18,27,29,30,35]	NMR and X-ray
**4**	14-*epi*-cochlioquinone B	C_28_H_4_0O_6_	Antibacterial activity; cytotoxic activity	*N. pura*	[15]	NMR and ROESY
**5**	*Epi*-cochlioquinone A	C_3_0H_44_O_8_	ACAT inhibition; cytotoxic activity	*S. bisbyi* SANK 17777	[16,20,26]	NMR and X-ray
**6**	Cochlioquinol	C_31_H_46_O_8_	Plant toxic activity	*B. cynodont*	[17,18,41]	NMR and X-ray
**7**	Cochlioquinones C	C_28_H_40_O_7_	Plant toxic activity; antibacterial activity; cytotoxic activity	*B. cynodontis cynA*	[17,18,19,20,21,22,23,24,25,26,29]	NMR
**8**	Cochlioquinones D	C_28_H_38_O_6_	Plant toxic activity; cytotoxic activity; antibacterial activity	*B. cynodontis cynA*	[18,21,23,26,27,29,30,32]	NMR
**9**	Cochlioquinones E	C_28_H_40_O_7_	Plant toxic activity; cytotoxic activity	*B. cynodontis cynA*	[18,27]	NMR
**10**	Cochlioquinol Ⅱ	C_31_H_48_O_8_	Plant toxic activity; inhibition of mitochondrial electron transfer	*B. cynodontis cynA*	[18]	NMR
**11**	Cochlioquinol Ⅲ	C_32_H_50_O_9_	Plant toxic activity; inhibition of mitochondrial electron transfer	*B. cynodontis cynA*	[18]	NMR
**12**	Cochlioquinone A1	C_33_H_50_O_9_	Inhibition of angiogenesis; DGK inhibition	*B. zeicola*	[19,45]	NMR
**13**	11-methoxycochlioquinone A	C_31_H_46_O_9_	Not reported	*B. Zeicola*; *S. chartarum*	[20]	NMR
**14**	12-*O*-methyl-*epi*-cochlioquinone A	C_31_H_46_O_8_	Cytotoxic activity	*B. Zeicola*; *S. chartarum*	[20]	NMR
**15**	Stemphones B	C_3_0H_42_O_9_	Antibacterial activity; cytotoxic activity	*Aspergillus* sp. FKI-2136	[21,23]	NMR
**16**	Stemphones C	C_3_0H_42_O_7_	Antibacterial activity; cytotoxic activity	*Aspergillus* sp. FKI-2136	[21,23,46]	NMR
**17**	Anhydrocochlioquinone A	C_3_0H_42_O_7_	Antinematodal activity; cytotoxic activity	*B. Oryzae*; *B. luttrelli*	[22,25]	NMR
**18**	Stemphone D	C_3_0H_42_O_8_	Antibacterial activity; cytotoxic activity	*Aspergillus* sp. FKI-2136	[23,45]	NMR
**19**	Stemphone F	C_33_H_48_O_9_	Antibacterial activity; cytotoxic activity	*Aspergillus* sp. FKI-2136	[23,24,45]	NMR
**20**	2,3-didehydro-19-hydroxy-14-*epi*-cochlioquinone B	C_38_H_38_O_7_	Antibacterial activity	*Nigrospora* sp. MA75	[24]	NMR
**21**	Cochilioquinone F	C_31_H_44_O_8_	Cytotoxic activity	*B. luttrelli*	[25]	NMR
**22**	*Epi*-cochlioquinone D	C_28_H_38_O_6_	Cytotoxic activity	*H. velutinum* TS28	[26]	NMR, NOE and ECD
**23**	12-α-hydroxy-*epi*-cochlioquinone D	C_28_H_38_O_7_	Cytotoxic activity	*H. velutinum* TS28	[26]	NMR, NOE and ECD
**24**	Cochlioquinone G	C_28_H_39_NO_6_	Cytotoxic activity	*B. sorokiniana* A606	[27]	NMR and NOE
**25**	Cochlioquinone H	C_28_H_38_O_6_	Cytotoxic activity	*B. sorokiniana* A606	[27]	NMR and ROESY
**26**	Arthripenoid D	C_30_H_44_O_10_	Not reported	*Arthrinium* sp. NF2194; *Nectria* sp. Z14-w	[28]	NMR, X-ray and HR-MS
**27**	Arthripenoid E	C_30_H_43_NO_9_	Not reported	*Arthrinium* sp. NF2194; *Nectria* sp. Z14-w	[28]	NMR, X-ray and HR-MS
**28**	Arthripenoid F	C_30_H_42_O_8_	Cytotoxic activity	*Arthrinium* sp. NF2194; *Nectria* sp. Z14-w	[28]	NMR, X-ray and HR-MS
**29**	Nectripenoid B	C_28_H_36_O_6_	Cytotoxic activity	*Arthrinium* sp. NF2194; *Nectria* sp. Z14-w	[28,30,32]	NMR, X-ray and HR-MS
**30**	Cochlioquinone I	C_28_H_38_O_7_	Cytotoxic activity	*Bipolaris* sp. L1-2	[29]	NMR and X-ray
**31**	Cochlioquinone J	C_29_H_42_O_7_	Cytotoxic activity	*Bipolaris* sp. L1-2	[29]	NMR and X-ray
**32**	Cochlioquinone K	C_29_H_42_O_8_	Cytotoxic activity	*Bipolaris* sp. L1-2	[29]	NMR and X-ray
**33**	Cochlioquinone L	C_29_H_40_O_8_	Cytotoxic activity	*Bipolaris* sp. L1-2	[29]	NMR and X-ray
**34**	Cochlioquinone M	C_30_H_40_O_7_	Cytotoxic activity	*Bipolaris* sp. L1-2	[29]	NMR and X-ray
**35**	Cochlioquinones N	C_30_H_45_NO_8_	Cytotoxic activity	*Bipolaris* sp. L1-2	[29]	NMR and X-ray
**36**	12-keto-cochlioquinone A	C_30_H_42_O_8_	Not reported	*B. sorokiniana* 11134	[30]	NMR and NOE
**37**	Cochlioquinone B derivative	C_22_H_30_O_7_	Antibacterial activity	*B. sorokiniana*	[31]	NMR
**38**	Furanocochlioquinone	C_28_H_34_O_6_	Cytotoxic activity	*C.* B5–2; *N. pseudotrichia* B69–1	[32]	NMR and ECD
**39**	Cochlioquinone-9	C_30_H_44_O_8_	Insect resistance activity; vasodilatory activity		[31,33,44]	NMR and HR-MS
**40**	Bipolariterpenes B	C_28_H_37_NO_5_	Not reported	*B. eleusines*	[34]	HR-MS, NMR and ECD
**41**	Bipolariterpenes C	C_28_H_39_NO_5_	Not reported	*B. eleusines*	[34]	HR-MS, NMR and ECD
**42**	Isocochlioquinone A	C_30_H_44_O_8_	Plant toxic activity; antiparasitic activity; antibacterial activity; cytotoxic activity	*B. bieolor* EI-1	[20,25,29,35,43]	NMR and X-ray
**43**	14-*epi*-dihydrocochlioquinone B	C_28_H_40_O_6_	Cytotoxic activity	*N. pura*	[15]	NMR
**44**	Isocochlioquinone C	C_28_H_40_O_7_	Plant toxic activity; antiparasitic activity; cytotoxic activity	*B. cynodontis* cynA	[18,22,23,25,27,29,30,43]	NMR
**45**	Isocochlioquinone A bis-acetyl derivative	C_32_H_46_O_9_	Antibacterial activity	*D. dematioideaz*	[23,36]	NMR
**46**	Stemphone E	C_30_H_44_O_9_	Antibacterial activity; cytotoxic activity	*Aspergillus* sp. FKI-2136	[23,45]	NMR and MS
**47**	Stemphones G	C_30_H_42_O_9_	Antibacterial activity; cytotoxic activity	*Aspergillus* sp. FKI-2136	[23,27]	NMR and MS
**48**	Isocochlioquinone B	C_30_H_42_O_8_	Not reported	*Cochliobolus* sp.	[23,30,37]	HR-MS and NMR
**49**	Isocochlioquinone D	C_30_H_43_NO_7_S	Cytotoxic activity	*B. sorokiniana* A606	[27,28]	HR-MS and NMR
**50**	Isocochlioquinone E	C_28_H_38_O_7_	Cytotoxic activity	*B. sorokiniana* A606	[27,28]	HR-MS and NMR
**51**	Arthripenoid A	C_31_H_45_NO_8_S	Not reported	*Arthrinium* sp. NF2194; *Nectria* sp. Z14-w	[28]	HR-MS, NMR and X-ray
**52**	Arthripenoid B	C_30_H_46_O_9_	Not reported	*Arthrinium* sp. NF2194; *Nectria* sp. Z14-w	[28]	HR-MS, NMR and X-ray
**53**	Arthripenoid C	C_30_H_44_O_9_	Inhibition of cona-induced T cell proliferation	*Arthrinium* sp. NF2194; *Nectria* sp. Z14-w	[28]	HR-MS, NMR and X-ray
**54**	Nectripenoid A	C_29_H_39_NO_6_S	Cytotoxic activity	*Arthrinium* sp. NF2194; *Nectria* sp. Z14-w	[28]	HR-MS, NMR and X-ray
**55**	Bipolahydroquinone A	C_28_H_42_O_7_	Cytotoxic activity	*Bipolaris* sp. L1-2	[29]	HR-MS, NMR and X-ray
**56**	Bipolahydroquinone B	C_28_H_38_O_7_	Cytotoxic activity	*Bipolaris* sp. L1-2	[29,39]	HR-MS, NMR and X-ray
**57**	Bipolahydroquinones C	C_28_H_40_O_7_	Cytotoxic activity	*Bipolaris* sp. L1-2	[29]	HR-MS, NMR and X-ray
**58**	Isocochlioquinones F	C_30_H_44_O_8_	Cytotoxic activity	*Bipolaris* sp. L1-2	[29]	HR-MS, NMR and X-ray
**59**	Isocochlioquinones G	C_28_H_40_O_7_	Cytotoxic activity	*Bipolaris* sp. L1-2	[29,38]	HR-MS, NMR and X-ray
**60**	19-dehydroxyl-3-*epi*-arthripenoid A	C_31_H_45_NO_7_S	Cytotoxic activity	*B. sorokiniana* 11134	[31,38]	MS and NMR
**61**	Δ12-19-dehydroxy-arhripenoid A	C_31_H_43_NO_6_S	Cytotoxic activity; cona-induced T cell proliferation	*B. zeicola*	[38]	NMR, X-ray and ECD
**62**	12,19-didehydroxy-arthripenoid A	C_31_H_45_NO_6_S	Cytotoxic activity; cona-induced T cell proliferation	*B. zeicola*	[38]	NMR, X-ray and ECD
**63**	Tetrahydrofuran-3-*epi*-cochlioquinone A	C_31_H_48_O_6_	Cytotoxic activity	*B. zeicola*	[38]	NMR, X-ray and ECD
**64**	Isotetrahydrofuran-3-*epi*-cochlioquinone A	C_31_H_48_O_8_	Cytotoxic activity	*B. zeicola*	[38]	NMR, X-ray and ECD
**65**	19-dehydroxy-arthripenoid A	C_31_H_45_NO_7_S	Cytotoxic activity; cona-induced T cell proliferation	*B. zeicola*	[38]	NMR, X-ray and ECD
**66**	Δ2-19-dehydroxy-arthripenoid A	C_31_H_43_NO_7_S	Cytotoxic activity; cona-induced T cell proliferation	*B. zeicola*	[38]	NMR, X-ray and ECD
**67**	4-acetoxy-isocochlioquinone D	C_32_H_47_NO_8_S	Cytotoxic activity; cona-induced T cell proliferation	*B. zeicola*	[38]	NMR, X-ray and ECD
**68**	4-acetoxy-31α-methoxy-isocochlioquinone D	C_33_H_49_NO_9_S	Cytotoxic activity	*B. zeicola*	[38,39]	NMR, X-ray and ECD
**69**	19-dehydroxyl-31-keto-3-*epi*-arthripenoid A	C_31_H_45_NO_8_S	Cytotoxic activity	*B. zeicola*	[38,39]	NMR, X-ray and ECD
**70**	Bipolaquinones A	C_30_H_44_O_6_	Cona induced T cell proliferation	*B. zeicola*	[39]	NMR, X-ray and ECD
**71**	Bipolaquinones B	C_30_H_44_O_6_	Cona induced T cell proliferation	*B. zeicola*	[39]	NMR, X-ray and ECD
**72**	Bipolaquinones C	C_28_H_42_O_5_	Not reported	*B. zeicola*	[39]	NMR, X-ray and ECD
**73**	Bipolaquinones D	C_28_H_44_O_5_	Not reported	*B. zeicola*	[39]	NMR, X-ray and ECD
**74**	Bipolaquinones E	C_28_H_44_O_5_	Not reported	*B. zeicola*	[39]	NMR, X-ray and ECD
**75**	Bipolaquinones F	C_30_H_46_O_7_	Cona induced T cell proliferation	*B. zeicola*	[39]	NMR, X-ray and ECD
**76**	Bipolaquinones G	C_30_H_46_O_7_	Cona induced T cell proliferation	*B. zeicola*	[39]	NMR, X-ray and ECD
**77**	Bipolaquinones H	C_30_H_46_O_7_	Cona induced T cell proliferation	*B. zeicola*	[39]	NMR, X-ray and ECD
**78**	Bipolaquinones I	C_30_H_46_O_7_	Cona induced T cell proliferation	*B. zeicola*	[39]	NMR, X-ray and ECD
**79**	Bipolaquinones J	C_28_H_42_O_6_	Cona induced T cell proliferation	*B. zeicola*	[39]	NMR, X-ray and ECD
**80**	Furanocochlioquinol	C_28_H_36_O_5_	Cytotoxic activity	*C. rosea* B5–2; *N. pseudotrichia* B69–1	[32]	HR-MS, NMR and ECD
**81**	Bipolaquinone K	C_28_H_36_O_6_	Cytotoxic activity; antioxidant activity	*B. sorokiniana*	[40]	NMR and MS

## Data Availability

No new data were created or analyzed in this study.
